# Spatiotemporal analysis and epidemiological characterization of the human immunodeficiency virus (HIV) in Libya within a twenty five year  period: 1993–2017

**DOI:** 10.1186/s12981-019-0228-0

**Published:** 2019-06-25

**Authors:** Mohamed A. Daw, Amina M. Daw, Nadia E. M. Sifennasr, Aisha M. Draha, Ahmed A. Daw, Ali A. Daw, Mohamed O. Ahmed, Ebtisam S. Mokhtar, Abdallah Hu. El-Bouzedi, Ibrahem M. Daw, Samia I. Adam, Saed Warrag, In association with Libyan Study Group of Hepatitis & HIV

**Affiliations:** 1 Department of Medical Microbiology & Immunology, Faculty of Medicine, University of Tripoli, CC 82668 Tripoli, Libya; 2 Department of General Medicine, Faculty of Medicine, University of Tripoli, CC 82668 Tripoli, Libya; 3 Department of Pharmacology, Faculty of Medicine, University of Tripoli, CC 82668 Tripoli, Libya; 4 Tripoli Medical Centre, Faculty of Medicine, Tripoli, CC 82668 Tripoli, Libya; 5 Department of Microbiology & Parasitology, Faculty of Veterinary Medicine, University of Tripoli, CC 82668 Tripoli, Libya; 6 Department of Laboratory Medicine, Faculty of Biotechnology, Tripoli University, CC 82668 Tripoli, Libya; 7 Department of Planning, Faculty of Engineering, University of Tripoli, CC 82668 Tripoli, Libya; 8 Department of Laboratory Medicine, Faculty of Biotechnology, Aljabel-Agarbi University, Nalot, Libya

**Keywords:** HIV/AIDS, Libya, Spatiotemporal analysis, Clusters, Demographic factors

## Abstract

**Background:**

Infection with the human immunodeficiency virus (HIV) is an alarming problem in North African countries, but few studies have analyzed the geographical distribution of the epidemic. Libya, the second largest country in Africa and with the longest coast on the Mediterranean basin facing Europe, has experienced major outbreaks of HIV infection. Since then, no studies have followed up on the burden of HIV infections. To plan interventions and allocate resources, spatial analysis of HIV/AIDS clusters are required in order to identify epidemic foci and trends in the country. The objective of this study was to assess HIV infection clustering and trends in Libya.

**Methods:**

Information on all recorded HIV/AIDS cases during 1993–2017 were extracted from the National Reporting System. A total of 8015 newly diagnosed HIV cases with address information were included. Spatial autocorrelation and spatial–temporal analysis were used to identify HIV clusters. Spatial correlations between cases and socio-demographic factors were determined using spatial regression.

**Results:**

HIV cases steadily increased within the Libyan population, particularly among those aged < 27 years. Spatiotemporal analysis showed marked geographic and temporal variation of HIV infection, particularly during 2005–2012. The risk factors varied from one region to another, and the contribution of injection drug use to infection increased with time. Four clusters in three time periods were identified, three on the Mediterranean coast and one in the south.

**Conclusion:**

HIV is an emerging problem in Libya, particularly among young adults. The infection rate varies greatly among the regions and districts, particularly within certain definable geographical areas. Effective intervention strategies are needed to contain HIV infections, especially within the endemic areas.

**Electronic supplementary material:**

The online version of this article (10.1186/s12981-019-0228-0) contains supplementary material, which is available to authorized users.

## Background

North Africa has been characterized as ‘‘a real hole in terms of HIV/AIDS epidemiological data.’’ It is estimated that half a million people are living with HIV/AIDS in North Africa, and the number of new infections is increasing [1, 2]. Different social, economic, demographic and even political factors are involved in the spread of HIV in the region. The contribution of the different factors varies greatly from one country to another and within the regions of the same country [3]. The spread of HIV is associated with high risk groups, including prisoners and injection drug users (IDUs), and recently it has been emerging among men who have sex with men [4, 5]. The political turmoil in the region and consequent influx of African immigrants has increased awareness and surveillance, as it has been shown that such instabilities can result in increased transmission and spread of infectious diseases, including HIV-1 infection [6, 7]. Several countries in the region still resist proceeding to second-generation surveillance systems that compile HIV serological and risk data from multiple sources [8].

Investigating the spatial distribution of an HIV epidemic can be challenging, particularly in sparsely populated countries. Monitoring the spread of HIV and identifying high-risk areas can provide useful information for planning to halt the spread of HIV and to provide better health services in the epidemic areas. Furthermore, it enables national healthcare policymakers to develop effective intervention strategies and allocate financial and human resources according to need [9, 10].

Libya is the second largest country in Africa but has the lowest population density. Sparsely populated large countries can mask geographical heterogeneity of HIV infection and may cause misinterpretation of survey data. A recent comprehensive study in Libya has shown that the prevalence of Hepatitis C Virus (HCV) varies geographically and has a variety of spatiotemporal patterns, with emergence of areas of high frequencies of HCV infection in specific regions and districts [11]. However, there is little information on the country-wide epidemiological distribution and spatial trends of HIV/AIDS. Moreover, the location of clusters and their correlation with socio-environmental factors remain unknown. Hence, understanding the factors associated with the geographic variation in HIV prevalence may also enable the targeted selection and application of the most suitable interventions. This has not been studied in Libya yet.

In Libya, the HIV surveillance system is based on mandatory, anonymous notification of newly diagnosed HIV cases by laboratories all over the country combined with epidemiological information on the mode of transmission and other clinical data, as reported by physicians and trained clinical epidemiologist. In 2008, the country carried out one of the largest population-based studies on the sero-prevalence of viral hepatitis and HIV in Africa. The study included over 1% of the total population according to the national census of 2003–2004 [12]. In 2014, another major cohort study was carried out on the sero-prevalence of HIV, HBV and HCV. These studies reported on the demographic variables and risk factors associated with the mode transmission of these viruses [13]. In 2017, we conducted a comprehensive study on the molecular and epidemiological characterization of HIV in Libya based on data collected over a period of 20 years. That study became a landmark for planning intervention programs targeting HIV-infected people in Libya [14].

In view of the paucity of information on the epidemiological trends and spatial distribution of HIV infections in Libya, this study was designed with three objectives in mind: (1) to describe the epidemiological and spatiotemporal distributions of HIV among the Libyan population; (2) to analyze the socio-demographic factors that could be influencing the spread of HIV; (3) to determine whether clusters of high or low HIV frequency exist in the country.

## Methods and data

### Study population

In 1992, the Libyan Study Group of Hepatitis & HIV, in cooperation with the Libyan Ministry of Health (formerly Secretariat of Health), established an automated registry of all cases of HIV/AIDS throughout the country. All the healthcare centers and institutions in the country were required to report identified HIV/AIDS cases. Thereafter, one of the largest national population-based surveillance studies in the world was conducted. The study included over 1% of the total Libyan population for HBV, HCV and HIV using the methodology described in previously published findings on the social and behavioral characteristics of the HIV-infected individuals registered in the HIV and hepatitis notification system (see 12879_2013_2969_MOESM1_ESM.doc in [12]). The information collected in that survey includes region of origin, gender, age, level of education, marital status, and other related risk and demographic factors. The HIV sero-status of all participants was tested and then confirmed by the enzyme-linked immunosorbent assay (ELISA). Samples that were positive on both tests were classified as positive [14].

The current study analyzed the data in the records that are restricted to persons diagnosed with HIV between 1993 and 2017 and with clearly identified addresses. Cases with incomplete information (n = 471) were excluded from the study. The geographic locations of those included in the study were accurately identified (Additional file 1: Figure S1 shows the Libyan regions and the districts within each region).

### Geospatial analysis

The geographic coordinates were collected using the geographic information system (GIS) and global positioning system (GPS) technologies. They were recorded at the center of the enumeration areas based on the geo-referenced information on the study participants. The corresponding national standard geo-codes at the provincial city and county levels were included in the analysis to identify the location of the reported cases. Population data at the provincial and county levels were obtained from the Libyan National Bureau of Statistics [15, 16]. The data were used to produce a map displaying clusters of low and high rates of HIV infection in the study area and depicted the variations of HIV rates in different regions in the country.

### Statistical analysis

Data were coded and entered into a database, which was then cleaned and verified [12]. Trends in demographic and behavioral characteristics among HIV/AIDS cases were analyzed by multivariate analysis to identify potential demographic and risk factors, using spatial-logistic regression with anti- HIV serologic results as the dependent variable (SPSS, Inc., Chicago, Illinois). The HIV estimates are reported with 95% confidence intervals (CI) determined by using the Poisson distribution approximation [13].

## Results

The demographic data of the 8015 identified cases of HIV infection with specific geographical data are shown in Table 1. Over 80% of the study population were < 40 years old, 55% were aged 21–30 years, and 26% were 31–40 years old. Most of infected individuals (4568; 57%) received no formal education, 28% (n = 2257) had primary school education. Over 85% (n = 6835) of the newly infected cases were single persons (p < 0.001).

**Table 1:** Demographic characteristics of the HIV-infected individuals included in the study

	Newly diagnosed cases of HIV n (%)				
	1993–1997	1998–2002	2003–2007	2008–2012	2013–2017
Region					
West	236 (8.1)	494 (16.9)	599 (20.5)	739 (25.3)	849 (29.1)
East	595 (21.9)	481 (17.7)	525 (19.3)	532 (19.6)	582 (21.4)
South	91 (7.0)	276 (21.7)	305 (23.9)	368 (28.9)	234 (18.5)
Central	53 (4.8)	81 (7.3)	206 (18.8)	287 (25.9)	482 (43.5)
Gender					
Male	758 (77.7)	1021 (76.7)	1220 (74.6)	1395 (72.4)	1650 (76.9)
Female	217 (22.3)	311 (23.3)	415 (25.4)	531 (27.6)	497 (22.3)
Age (years)					
< 20	58 (5.9)	77 (5.8)	123 (7.5)	137 (7.1)	119 (5.5)
21–30	617 (63.3)	744 (55.9)	80 (4.9)	995 (51.7)	1221 (56.9)
31–40	210 (21.5)	328 (24.6)	479 (29.3)	509 (26.4)	549 (25.6)
41–50	76 (7.8)	102 (7.7)	139 (8.5)	176 (9.1)	161 (7.5)
> 50	14 (1.4)	81 (6.1)	93 (5.7)	109 (5.7)	97 (4.5)
Education					
No formal education	503 (51.6)	728 (54.7)	991 (60.6)	1133 (58.8)	1213 (56.5)
Primary School	312 (32)	390 (29.3)	418 (25.6)	527 (27.4)	610 (28.4)
Secondary	149 (15.3)	197 (14.8)	211 (12.9)	249 (12.9)	311 (14.5)
Superior	11 (1.1)	17 (1.3)	15 (0.90)	17 (0.9)	13 (0.6)
Marital status					
Single	786 (80.6)	1113 (83.6)	1392 (85.1)	1667 (86.6)	1877 (87.4)
Married	13 (1.3)	16 (1.2)	21 (1.3)	19 (1.0)	23 (1.1)
Divorced	97 (9.9)	109 (8.2)	113 (6.9)	123 (6.4)	120 (5.6)
Unknown	79 (8.1)	94 (7.1)	109 (6.7)	117 (6.1)	127 (5.9)
Total	975 (12.2)	1332 (16.6)	1635 (20.6)	1926 (24.0)	2147 (26.8)

The overall proportion of HIV cases increased from 12.2% (n = 975) in 1993 to 26.8% (n = 2147) during 2013–2017. Young males had the highest rates of HIV throughout the study period, reaching up to 75%. However, in females it was steady, ranging from 22 to 27% with no significant increase during the study period.

**Fig. 1:**
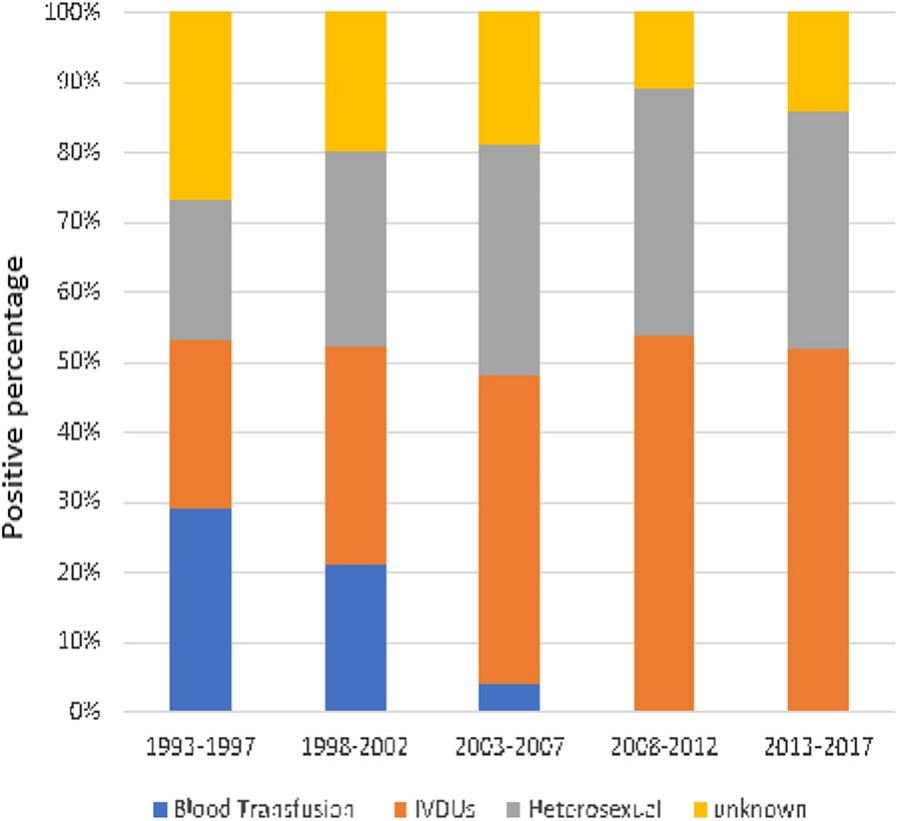
Analysis of risk factors associated with the spread HIV in Libya during 1993–2017. *IDUs* injection drug use

Figure 1 shows the risk factors influencing the rate of HIV. The proportions of individuals infected via blood or blood products were high (20–30%) during 1993–1997 and then decreased to 5% by 2003–2007, after which no cases were reported. Sexual promiscuity was associated with 20% of the infected cases during 1993–1997, after which the proportion increased steadily to reach 40% during 2013–2017. The proportion of IVDUs increased significantly over time *(*p < 0.001): it was 20% during 1993–1997 and reached over 50% during 2008–2017.

The national and regional trends of HIV infection were analyzed during the study period (Additional file 1: Figure S2). The eastern region had the highest HIV rate in comparison to other regions during 1993–1997 (9.8%) but narrowing to a 1.1% gap between 1998 and 2002, and then declined steadily to < 4% the national rate. The next highest rate was in the western region, where it ranged between 8 and 29% (p < 0.001). The central and the southern regions had the lowest rates of HIV during 1993–2008.

The number of HIV cases in the Libyan provinces is shown in Table 2. Logistic regression analysis showed that the HIV infection rate differed significantly from one province to another, even within the same region. HIV infections were more frequent in 11 provinces: Tripoli, Benghazi, Darna, Al-butnan, Musrata, Anniqat Al-khams, Al-Jabal Al-gharbi, Nalut and Azzawia.

**Table 2:** Regional and provincial distribution of HIV infections and in Libya, 1993–2017

Region/district	No. (%)	OR	95% CI
Western region			
Tripoli	1191 (14.9)	8.75	1.5–19.7
Az-zawua	394 (4.9)	6.93	1.4–14.97
An-nuqat Al-chams	439 (5.5)	7.03	1.10–16.78
Nalut	397 (5.0)	6.61	1.12–13.17
Al-Jabal Al-gharbi	496 (6.2)	7.97	1.01–1571
Central region			
Surt	130 (1.6)	1.01	0.12–3.32
Musrata	406 (5.1)	7.19	1.52–16.91
Zleitan	201 (2.5)	2.1	1.02–4.97
Al-Jufra	187 (2.3)	2.26	1.11–3.87
Al-murgub	185 (2.3)	4.31	1.49–7.43
Eastern region			
Benghazi	1211 (15.1)	9.17	1.86–20.36
Al-wahat	318 (4.0)	4.03	1.81–9.38
Al-Jabal Al-achdar	332 (4.1)	3.21	1.09–7.03
Darna	443 (5.5)	6.19	1.92–14.02
Al-butnan	411 (5.1)	5.79	1.99–13.02
Southern region			
Sabha	317 (4.0)	6.07	1.76–15.19
Wadi-alhaya	273 (3.4)	3.12	0.98–6.67
Wadi Ascha-schati	259 (3.2)	3.09	1.05–6.01
Ghat	209 (2.6)	2.87	1.32–5.01
Murzuq	216 (2.7)	2.96	0.97–4.19

**Fig. 2:**
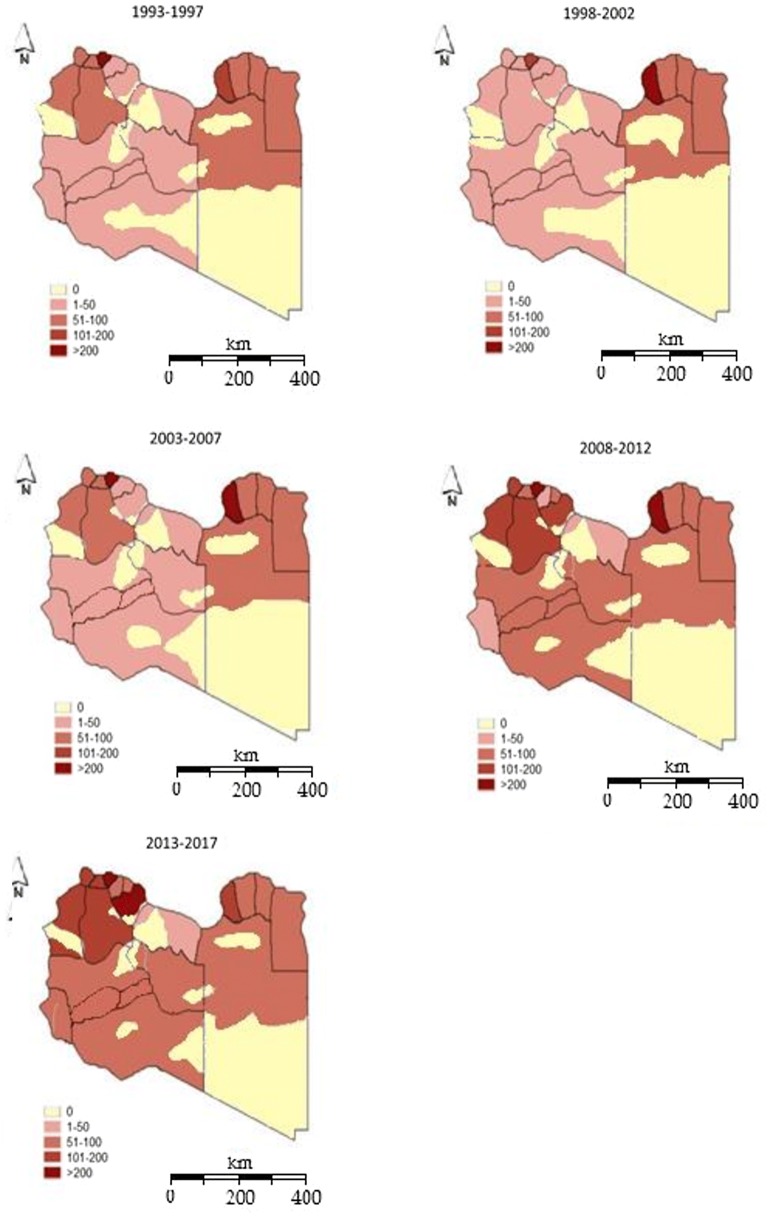
Geographic distribution of reported HIV cases at the district level in Libya during 1993–2017

The HIV rates were analyzed by geographic location for each 5-year period. The HIV rates varied from one place to another regardless of provincial boundaries (Fig. 2). HIV intensity decreased gradually between 1993 and 2007 but then generally increased until it reached the highest level in 2017, with emergence of new epicenters particularly in the central and southern regions.

During 1993–1997, a larger number of HIV infections was observed in the eastern region, particularly in Benghazi this was associated with Bulgarian Nurses incident at Aljala Paediatric hospital. By contrast, HIV intensity was lower in the central, western and southern regions. From 1998 onward, there was an increase in the number of new cases of HIV all over the country, particularly in the west, center and south. During 1998–2002, the number of HIV cases increased particularly in the provinces of Anniqat Al-khams, Al-Jabal Al-gharbi, Nalut, and Azzawia in the western region but remained steady in the other three regions. The highest number of infections was reported in Musrata in the central region and in Sabha in the south.

**Fig. 3:**
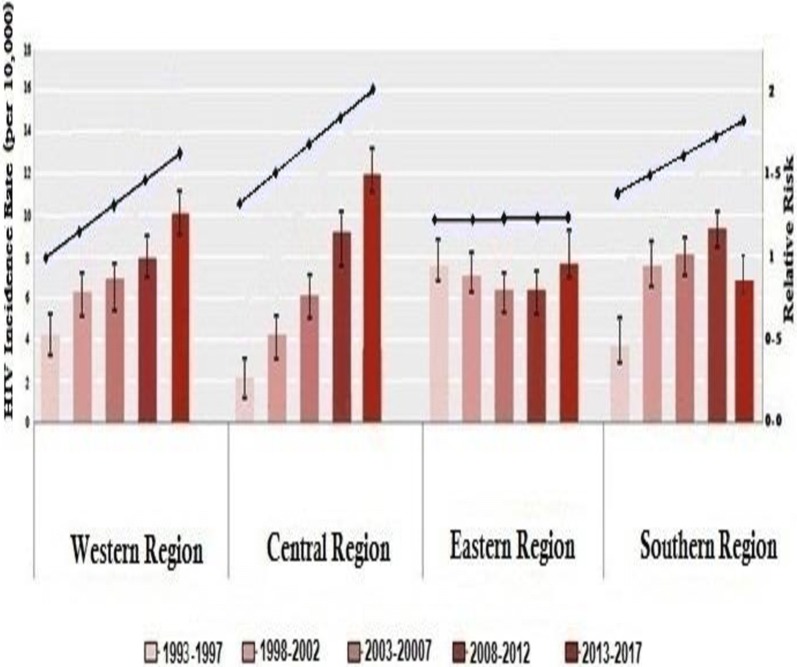
Temporal trends of estimated HIV incidence rate per 10,000 person-years (columns) and relative risk (blue lines) in the four Libyan regions during 1993–2017

Figure 3 illustrates the impact of attributable relative risk (RR) factors on the endemicity of HIV in each region. The RR for HIV-infected individuals in the western region was 1.31 (95% CI 0.86–1.51) in 1993–1997 and rose to 1.79 (95% CI 1.65–2.13) in 2013–2017, with a slight increase over the study period (*p* = 0.001). In the eastern region, the RR was relatively high during 1992–1997, as it reached 1.7 (95% CI 1.51–1.96). Then it remained steady and was 1.51 (95% CI 1.32–1.77) during 2013–2017. The RR of infection in the central and southern regions increased remarkably during the study period. It was 0.47 (95% CI 0.31–0.81) in 1993–1997 and rose to 1.92 (95% CI 1.85–2.32) in 2013–2017. The RR in the southern region was 0.51 (95% CI 0.31–0.86) in 1993–1997 and rose to 1.52 (95% CI 1.35–2.12) in 2013–2017. When RR was compared among the four regions, it was found to be significantly higher in the central and southern regions, particularly during 2013–2017 (*p* < 0.001).

**Fig. 4:**
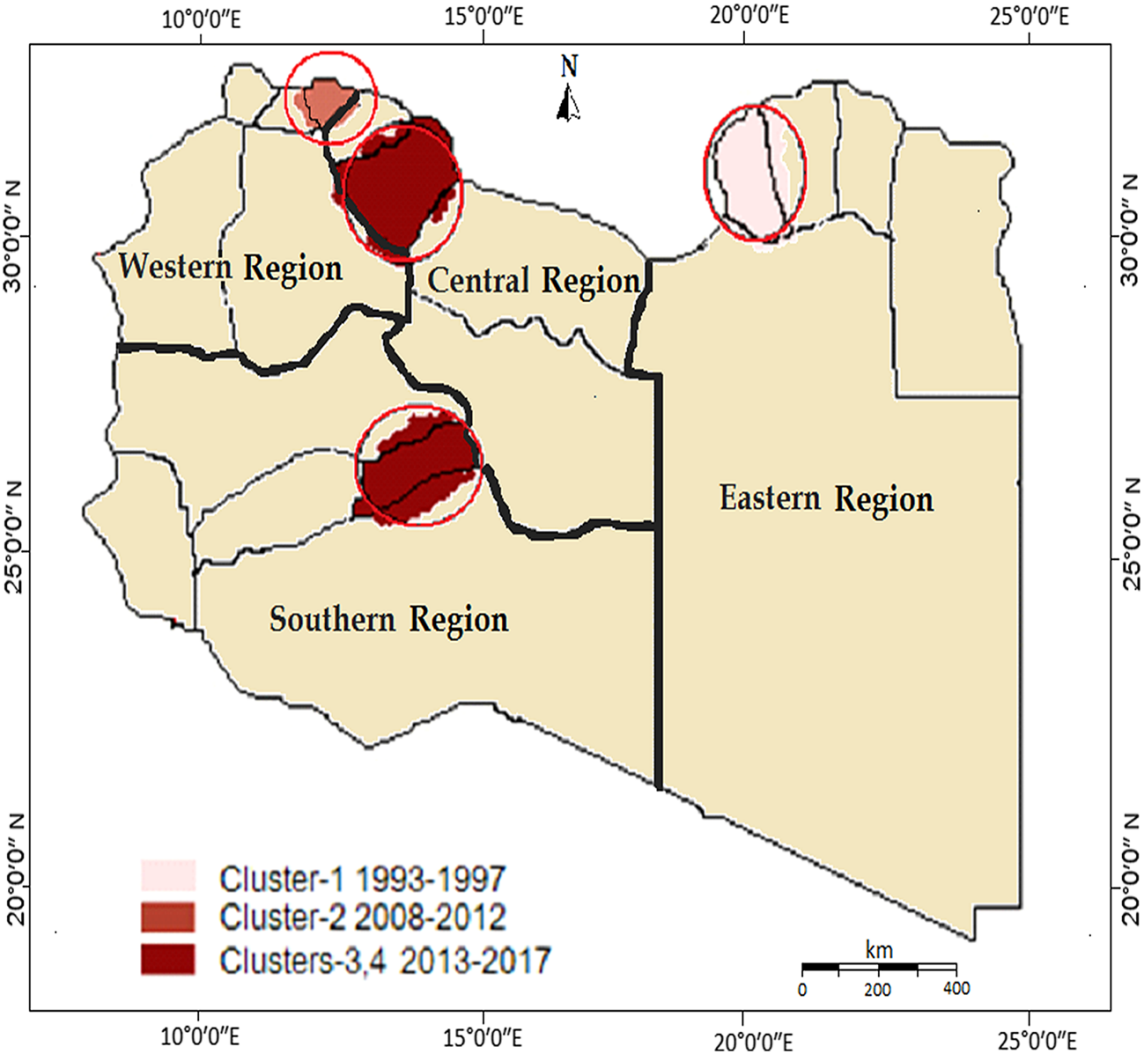
Distribution of high frequency spots of HIV infections in Libya between 1993 and 2017

Four clusters of high HIV rates occurring at different times and in different areas were identified. These clusters included 1287 cases (16% of all) (Fig. 4). The epidemiological and geographical characteristics of the clusters are shown in Table 3. The first cluster appeared during 1993–1997 and included 361 (32%) of the HIV cases mainly children who were deliberately infected. It covered Aljala and Alhawary districts of Benghazi, the largest city in the eastern region. It was centered at LLR = 32°06′53″N, 20°04′06″E, 21 m (68 ft), radius = 6.03 km (RR = 3.44). Compared to neighboring districts, the cases identified in this cluster had a 3.4 times higher HIV risk. The second cluster occurred during 2008–2012 and consisted of 203 cases (7% of all HIV cases). It was located in Tripoli in the western region at LLR = 32°52′30″N, 13°11′14″E, 21 m (68 ft), radius = 8.75 (RR = 5.02). It was spread over two districts, mostly involving the Old City and Soug Aljuma in Tripoli, the most heavily populated city in the western region. The third cluster was reported in Musrata (largest in the central region) and consisted of 406 HIV cases (36.6% of total) detected between 2013 and 2017 at LLR 32°37′75″N, 15°9′20″E, 10 m (3 ft), radius = 7.05 km (RR = 7.15). It covered the main district of Aldafnyia and Tripoli area Street in the city of Musrata. The fourth cluster was detected in Sebha between 2013 and 2017 and consisted of 317 (25.4%) HIV cases at LLR 27°02′15″N, 14°25′41″E, 421 m (1381 ft), radius = 10.27 (RR = 6.32, p = 0.001), covering Sabha city, the largest in the south.

**Table 3:** Spatial and temporal clusters of HIV infection and their relative risk in Libya 1993–2017

Cluster no.	Years	Coordinates		Elevation		Radius	Relative risk	*p* value
		North	East	m	ft	km		
1	1993–1997	32°06′53″	20°04′06″	21	68	9.03	3.44	0.001
2	2008–2012	32°52′30″	13°11′14″	21	68	8.75	5.02	0.001
3	2013–2017	32*°*37*′* 75*″*	15*°*09*′*20*″*	10	30	7.05	7.15	0.001
4	2013–2017	27°02′15″	14°25′41″	421	1381	14.27	6.32	0.001

## Discussion

This study indicates that Libya had a substantial number of HIV cases during 1993–2017. Different factors could have contributed to the increase of HIV cases in the country. Such factors varied from one region to another and over time. The geospatial analyses have shown great variability in the distribution of HIV cases within the country. However, vacant areas with no HIV cases were reported particularly within the Middle and Saharan areas. The highest rate of HIV, reported in the eastern region during 1993–2002, was particularly associated with Bulgarian Nurses Incident and other iatrogenic factors. From 2003 onwards, the incidence of HIV increased up to three folds in the central and southern regions. Logistic analysis showed that HIV/AIDS was more frequent in certain provinces within the regions themselves. The relative risk also varied with time and place. The highest RR was reported between 2008 and 2017, particularly in the central and southern regions and was associated with the emergence of new risk factors, including IVDU. These factors could have influenced the geographical distribution and local dynamics of the HIV epidemic. Similar results have also been reported in other African countries, including Burundi and South Africa [17, 18].

A variety of demographic and risk factor have been identified in the transmission of HIV in Libya. The main predisposing factors were IVDU and heterosexual activities, particularly in recent decades. This indicates that societal shifts and behavioral patterns have increased the vulnerability to HIV infection. Similar upsurges of HIV epidemics have also been reported in other countries [9, 10]. The highest number of adolescents living with HIV has been reported in sub-Saharan Africa, followed by Southeast Asia, the Pacific, and Latin America [19–21]. This supports a rising awareness that HIV testing among young people should be encouraged, and that easy access to youth-friendly counseling centers should be provided.

Our analysis shows that the HIV epidemic exists as spatially defined sub-epidemics at regional and districts levels. This allowed us to identify populations at higher risk of HIV infection due to geographic, social, education or behavioral factors. Areas of highly prevalent HIV should be monitored so that more effective prevention strategies may be applied as needed [22, 23].

Our study identified four clusters of high HIV infection rates, three of which are on the Mediterranean coast (Benghazi, Tripoli and Musrata) and the fourth in Sabha in the middle of the Libyan desert, neighboring sub-Saharan African countries. Evidence from earlier studies revealed that Benghazi was the only province that showed significant clusters of plasma recipients. While the HIV epidemic in Tripoli and Sabha were initially driven by IDU and large numbers of African immigrants from Sub-Saharan Africa, in Musrata it was associated with IVDU and homosexuality [24–26]. These findings clearly indicate the need for ensuring sufficient access to good quality health services in these areas. We recommend that more attention and health resources should be directed to such districts, and studies are needed to examine the reasons for this increase.

The study has some limitations that need to be considered when interpreting the results. As this was a record-based study, some HIV/AIDS cases might not have been reported, which could have influenced the results of the analysis, particularly in the hot-spot areas. However, the study has generated important insights into the dynamics of HIV transmission among Libyan populations and provides visual and quantitative descriptions of the geographic characteristics of HIV infections in areas where infectious diseases are known to be under-reported [27, 28].

Investigating the geographical distribution of HIV infection in sparsely populated large areas such as Libya is challenging, but it is important for public health strategies. Such studies enable targeting surveillance and preventive or remedial measures to areas where the infection is clustered. They also urge the authorities to implement the necessary measures [29, 30].

## Conclusion

This study analyses the geographic and spatiotemporal distribution of HIV infection in Libya and highlights the risk factors involved. To the best of our knowledge, this is the first study in North Africa and the Arab region to characterize the spatiotemporal epidemiology of HIV infection. Our findings showed clear variation in the distribution of HIV infection at the regional and district levels during 1993–2017. This indicates that intervention strategies for HIV prevention should be focused on particular areas or high-risk groups, as a national policy may not be the most efficient. In Libya, HIV infections were particularly more prevalent in the eastern and southern regions. Nevertheless, it should be noted that Central Libya has seen a sharp increase in recent years. This warrants more resources to prevent the resurgence of AIDS in this area. This could have been due to the increasing number of African immigrants and population movements in these regions as our previous work showed [7, 25]. Further studies are needed at the regional level in North African and Sub-Saharan countries bordering Libya, particularly Egypt, Sudan, Chad and Niger, to shed light on the population-related determinants that may play a role in the upsurge of HIV and other concomitant infections in North Africa [31, 32].

## Additional file

**Additional file 1: Table S1.** Libyan regions, districts, administrative boundaries, and population density. **Figure S1**: Map showing division of Libyan regions and districts covered by the study. **Figure S2**: National and regional trends of HIV infection in Libya 1993–2017.

## Data Availability

The data presented in this paper are freely available upon request.
