# Supplementary material for: Spatiotemporal analysis and epidemiological characterization of the human immunodeficiency virus (HIV) in Libya within a twenty five year period: 1993–2017
Source: AIDS Res Ther. 2019 Jun 25;16:14. doi: 10.1186/s12981-019-0228-0 (PMC6591977; doi:10.1186/s12981-019-0228-0)
Supplement: Supplementary file 1 — Additional file 1: Table S1. Libyan regions, districts, administrative boundaries, and population density. Figure S1: Map showing division of Libyan regions and districts covered by the study. Figure S2: National and regional trends of HIV infection in Libya 1993–2017. [file 12981_2019_228_MOESM1_ESM.docx]

**Additional file -1**

**Figure S1.** The geo-location of Libyan regions and districts involved in the study

**Table S1**; Libyan regions, Districts, administrative boundaries and population density

**Figure S2; National and Regional trends of HIV infection in Libya 1993 2017**


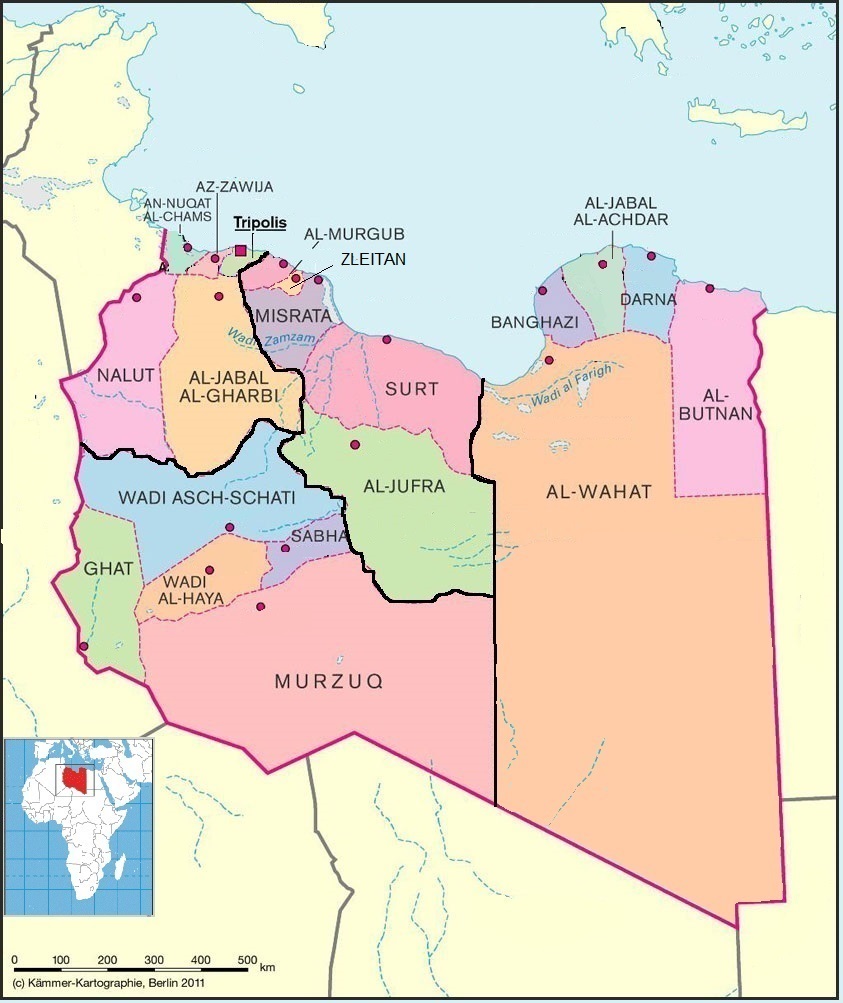


**Figure-S1.** The geo-location of Libyan regions and districts involved in the study

**Table S1**; Libyan regions, Districts, administrative boundaries and population density

| **District (Arabic)** | **District (English)** | **Area (km^2^)** | **Population** | **Number** |
| --- | --- | --- | --- | --- |
| **East Region** | | | | |
| البطنان | [Butnan](https://en.wikipedia.org/wiki/Butnan_District) | 84,996 | 159,536 | 1 |
| درنة | [Derna](https://en.wikipedia.org/wiki/Derna_District) | 31,511 | 163,351 | 2 |
| الجبل الأخضر | [Jabal al Akhdar](https://en.wikipedia.org/wiki/Jabal_al_Akhdar) | 11,429 | 203,156 | 3 |
| بنغازي | [Benghazi](https://en.wikipedia.org/wiki/Benghazi#Administrative_divisions) | 11,372 | 856,645 | 4 |
| الواحات | [Al Wahat](https://en.wikipedia.org/wiki/Al_Wahat_District) | 539,134 | 227,151 | 5 |
| **Central Region** | | | | |
| سرت | [Sirte](https://en.wikipedia.org/wiki/Sirte_District) | 225,437 | 193,720 | 6 |
| الجفرة | [Jufra](https://en.wikipedia.org/wiki/Jufra_District) | 117,410 | 52,342 | 7 |
| مصراتة | [Misrata](https://en.wikipedia.org/wiki/Misrata_District) | 29,172 | 550,938 | 8 |
| زليتن | Zleitan | 21,231 | 120,872 | 9 |
| المرقب | [Murqub](https://en.wikipedia.org/wiki/Murqub_District) | 6,796 | 432,202 | 10 |
| **West Region** | | | | |
| طرابلس | [Tripoli](https://en.wikipedia.org/wiki/Tripoli_District,_Libya) | 3,501 | 1,5186,13 | 11 |
| الزاوية | [Zawiya](https://en.wikipedia.org/wiki/Zawiya_District) | 2,753 | 290,993 | 12 |
| النقاط الخمس | [Nuqat al Khams](https://en.wikipedia.org/wiki/Nuqat_al_Khams) | 6,089 | 287,662 | 13 |
| الجبل الغربي | [Jabal al Gharbi](https://en.wikipedia.org/wiki/Jabal_al_Gharbi_District) | 76,717 | 304,159 | 14 |
| نالوت | [Nalut](https://en.wikipedia.org/wiki/Nalut_District) | 67,191 | 93,224 | 15 |
| **South Region** | | | | |
| وادي الشاطئ | [Wadi al Shatii](https://en.wikipedia.org/wiki/Wadi_al_Shatii_District) | 97,160 | 78,532 | 16 |
| سبها | [Sabha](https://en.wikipedia.org/wiki/Sabha_District) | 107,310 | 134,162 | 17 |
| وادي الحياة | [Wadi al Hayaa](https://en.wikipedia.org/wiki/Wadi_al_Hayaa_District) | 31,485 | 76,858 | 18 |
| غات | [Ghat](https://en.wikipedia.org/wiki/Ghat_District) | 68,482 | 23,518 | 19 |
| مرزق | [Murzuq](https://en.wikipedia.org/wiki/Murzuq_District) | 356,308 | 78,621 | 20 |

**Figure S2; National and Regional trends of HIV infection in Libya 1993-2017**

**
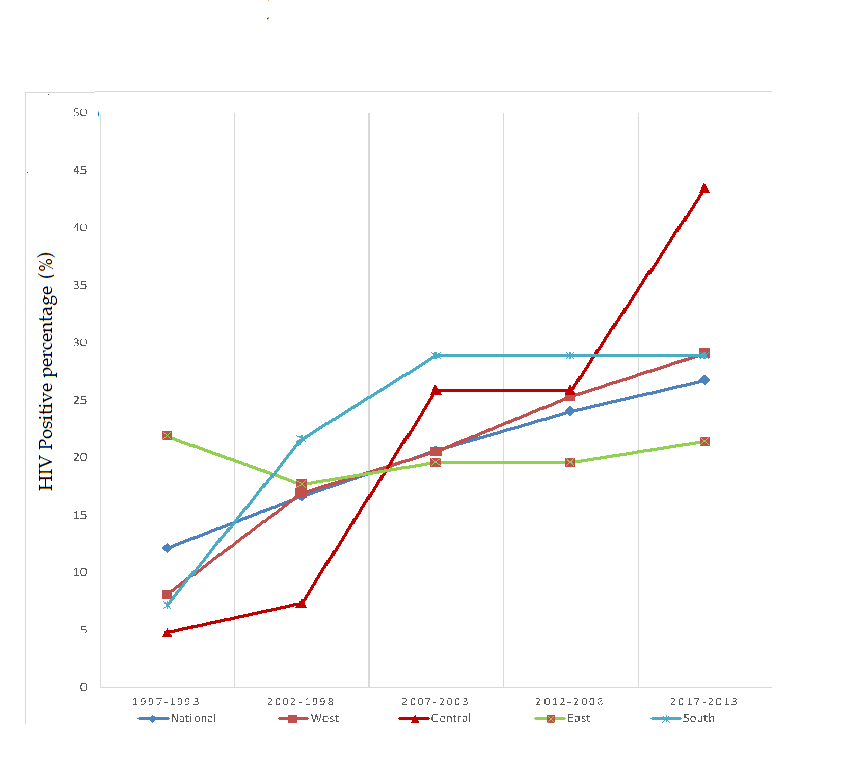
**
